# Moiré Energy Dissipation Driven by Nonlinear Dynamics

**DOI:** 10.1021/acsnano.4c16817

**Published:** 2025-04-30

**Authors:** Shuyu Huang, Yiming Song, Antoine Hinaut, Gema Navarro-Marín, Yunfei Chen, Ernst Meyer, Thilo Glatzel

**Affiliations:** † Key Laboratory for Design and Manufacture of Micro-Nano Biomedical Instruments, School of Mechanical Engineering, Southeast University, Nanjing 211189, China; ‡ Department of Physics, University of Basel, 4056 Basel, Switzerland; § Institute for Applied Physics, Justus Liebig University Giessen, 35392 Giessen, Germany

**Keywords:** phononic dissipation, friction force, noncontact AFM, contact AFM, Moiré superlattice, graphene

## Abstract

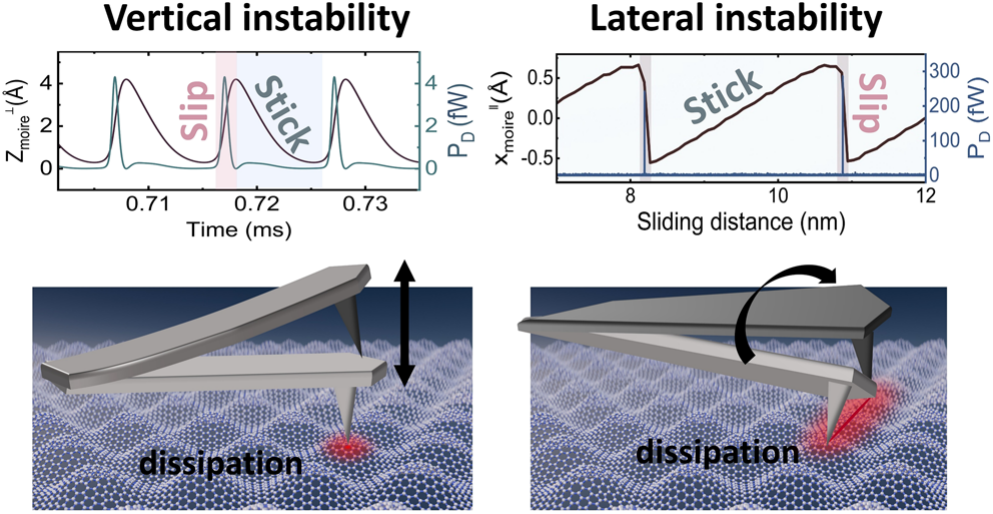

The moiré superlattice in twisted van der Waals heterostructures is of central importance for the modulation of the electronic and optical properties of the system, yet the mechanical dissipation of such moiré systems remains largely unexplored. Here, we report the experimental observations of energy dissipation across both vertical and lateral directions along the moiré superstructures, revealing a significant increase in dissipation at moiré ridges compared to flat domains. Comparison of the measurements with a theoretical phononic dissipation model suggests that the local increase in energy dissipation originates from nonlinear instability dynamics of the moiré superstructure. Criteria for such moiré energy dissipation are established, which are expected to be broadly applicable to other van der Waals heterostructures. Our results extend the understanding of mechanical energy loss in moiré systems and support the rational design of slidtronic and twisttronic devices and nanoelectromechanical systems in general.

## Introduction

Moiré superlattices, arsing from the overlay of two periodic structures with slightly mismatched lattices, have recently emerged as a fascinating platform for exploring a variety of novel physical phenomena. Although the extraordinary electronic, optical and nonlinear physics^1−5^ of moiré structures have been widely explored, their intriguing mechanical response and energy dissipation^6,7^ are not well understood. This gap in understanding material properties is hampering advances in energy and thermal management for nanodevices, potentially impacting the lifetime and performance of next-generation electronic devices, even quantum computers.

The energy dissipation in moiré superlattices can be thoroughly characterized by various advanced spectroscopic and microscopic techniques. Techniques such as Raman spectroscopy^8,9^ and infrared nanoimaging^10−12^ allow for assessing vibrational dynamics and thermal properties, providing indirect insights into energy dissipation by analyzing spectral shifts. In a more direct approach, atomic force microscopy (AFM) enables precise mapping of energy dissipation across the surface. Of these methods, AFM provides the most direct and localized access to energy dissipation, a feature indispensable for optimizing the design and function of devices incorporating moiré superstructures. Additionally, unlike conventional two-dimensional materials, the structural complexity of moiré superlattices extends into three dimensions, where energy dissipation is expected to occur both vertically and laterally, complicating the understanding of their mechanical responses and adding a critical layer of depth to their study.

Friction force microscopy (FFM), as a widely used contact mode AFM, allows the investigation of the energy dissipation of moiré structures during lateral sliding motions. Recent studies revealed stick–slip dynamics at the moiré-level of graphene superlattices on a metal surface.^13−15^ Such dual-scale stick–slip exists in the moiré system in van der Waals (vdW) heterostructures of two-dimensional (2D) materials, as well.^16−18^ The transition between superlubric and dissipative sliding has already been observed in a graphene/Pt system, which is governed by the moiré flexibility that can be tuned by the moiré size, the external load and the sliding velocity.^13,14^ Previous studies on moiré dissipation have focused on lateral dissipation using FFM, revealing the in-plane behavior and its implications on superlubricity. In contrast, little attention has been paid to vertical dissipation, which provides insights into out-of-plane interactions and the elastic deformation of atomic layers.^19−21^

Noncontact atomic force microscopy (NC-AFM) enables precise measurement of vertical dissipation. In this mode, the cantilever oscillates vertically at its resonance frequency with constant amplitude above the surface and measures the dissipated energy in the attractive force regime. Taking advantage of this technique, researchers have studied vertical energy dissipation at the atomic scale in various systems,^22−27^ but have rarely focused on dissipation specifically within the moiré superstructure.^19,20^

In this work, we comprehensively investigate for the first time the mechanical dissipation between the scanning probe and the graphene/Ir(111) moiré surface across both in-plane and out-of-plane directions providing a quantitative comparison. This analysis was enabled by our home-built ultrahigh vacuum AFM combining noncontact and contact modes.^28^ In addition, a dynamic force microscopy (DFM) model was newly developed to simulate the motions of the tip and the moiré surface during NC-AFM measurements. This innovative model incorporates the out-of-plane stiffness of the moiré superstructure, significantly enhancing our understanding of the energy dissipation mechanisms observed in experiments. Laterally, a two-state phononic friction (PF) model, that accounts for the in-plane elastic deformation of the moiré, was used to understand the mechanism of lateral dissipation during sliding at atomic- and moiré-levels and to explain our further observations of frictional load and velocity dependence.

## Results

**Figure 1:**
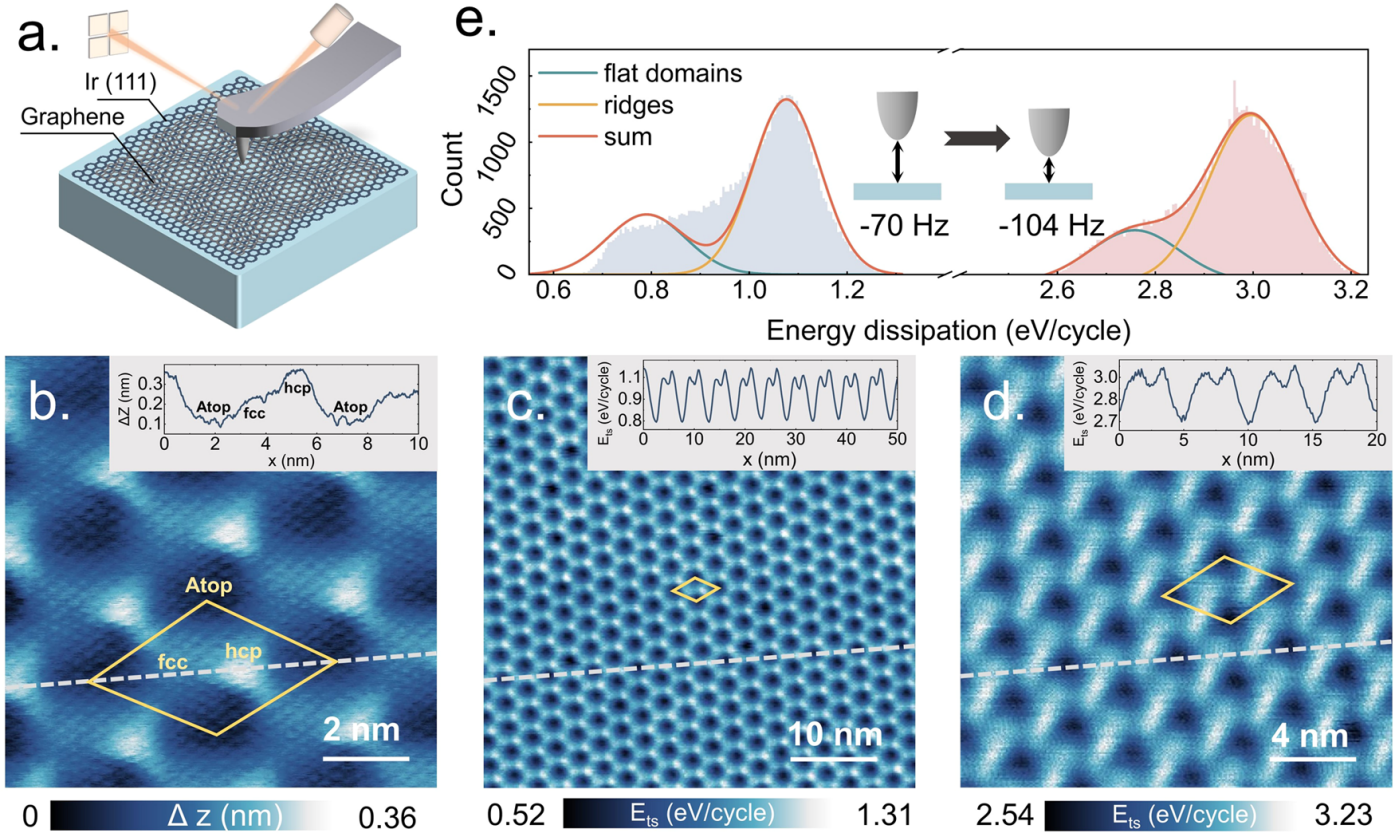
(a) Schematic drawing of the experimental setup with an AFM tip scanning over the Gr/Ir(111) moiré superlattice. (b) Atomically resolved morphology of the moiré superlattice characterized by NC-AFM, the three high-symmetry domains ATOP, FCC, and HCP are indicated. (c) Dissipation of the moiré superlattice at a frequency shift Δ*f* = −70 Hz, and (d) of −104 Hz. (e) Histograms of the dissipation distributions fitted to Gaussian functions obtained from the dissipation maps in (c, d). Measurement parameters: *A*_1st_ = 4 nm in (b–d), Δ*f* = −7 Hz in (b). The insets in (b–d): line profiles corresponding to the white dashed lines.

Monolayer graphene (Gr) with a moiré superlattice was formed on a Ir(111) surface (see Supporting Information (SI) for further details on sample preparation). The specific growth temperature and the natural lattice mismatch between the two materials resulted in only one single moiré pattern.^29^ We characterized the moiré superstructure with NC-AFM at room temperature (see Figure 1(a)), showing an atomically resolved hexagonal graphene lattice and a moiré superlattice (Figure 1(b)) with periodicities of 0.25 ± 0.01 and 2.71 ± 0.12 nm, respectively. In the moiré superstructure, the three high-symmetry domains ATOP, FCC, and HCP are observed within the moiré pattern, and the out-of-plane corrugation of the moiré supercell is ∼250 pm, as shown in the line profile in Figure 1(b). The Abbreviations refer to the local stacking configurations: FCC (face-centered cubic), HCP (hexagonal closed packed) and ATOP, where the carbon atoms are on top of the Iridium atoms. For clarity, in the following text, we refer to the ATOP region as the “moiré flat domain” and the FCC and HCP regions together as “moiré ridges”.

In NC-AFM measurements, the energy dissipation resulting from the tip–sample interaction can be investigated simultaneously with the topography. Considering the tip–sample interaction, vdW forces are the key factor that dominates the dynamic process and dissipation of the system. To maintain a constant oscillation amplitude of the cantilever, the driving excitation voltage is adjusted to compensate for the dissipation. Thereby the noncontact energy dissipation *E* per oscillating cycle can be determined from the driving excitation voltage, as previously reported.^30,31^ This dissipation depends on the distance between tip and sample, which can be modulated by the frequency shift Δ*f*; a more negative value corresponds to a closer tip–sample distance. The resulting vertical tip–sample dissipation (*E_ts_*) and its spatial distribution across the moiré superstructure are shown in Figure 1(c,d) for different tip–sample distances. Further details regarding the dissipation calculation are provided in Section 2 of the SI. Both measurements consistently reveal that the dissipation at the moiré ridges is increased compared to the flat domains.

When the tip oscillates above the surface at a relatively large distance (Δ*f* = −70 Hz), a higher dissipation is measured at the moiré ridges with an approximate energy loss per oscillation cycle of the cantilever of *E*_1,*r*_^⊥^ = 1.08 eV/cycle, while a lower value is found at the flat moiré domains of *E*_1,*f*_^⊥^ = 0.79 eV/cycle, as shown in the line profile of Figure 1(c). Since the energy loss arises from the interaction between the tip and the surface, the dissipation simply increases with reducing the tip–sample distance (Δ*f* = −104 Hz). The resulting dissipation map (Figure 1(d)) again shows a similar contrast between the moiré ridges and the flat areas. The line profile in Figure 1(d) shows that the energy dissipation increased to *E*_2,*r*_^⊥^ = 3.00 eV/cycle and *E*_2,*f*_^⊥^ = 2.75 eV/cycle at ridges and flat domains, respectively. In order to determine the contributions of moiré ridges and flat domains to the energy dissipation, we statistically analyzed the distributions of energy dissipation from Figure 1(c,d). The histograms are shown in Figure 1(e), where gray and pink bars represent the data extracted from Figure 1(c,d), respectively. Both histograms exhibit two distinct local maxima which can be fitted by a sum of two Gaussian distributions. The peaks at 0.79 and 1.08 eV/cycle in the gray histogram are attributed to dissipation arising from flat domains (yellow line) and ridges (green line), respectively. However, they collectively shift toward higher dissipation (see pink histogram) as the tip–surface interaction increases. Thereby, the dissipation peaks increased to 2.75 eV/cycle for flat domains and 3.00 eV/cycle for ridges. The dissipation histograms show that the height and position of the peaks are dominated by the ridges, regardless of whether the tip is close to or far from the sample. Our results demonstrate that the energy dissipation increases with decreasing tip–sample distance, which is consistent with previous studies and was expected.^25,30^ It is noteworthy that Figure 1(d) exhibits a remarkable broadening deformation of the moiré structure along the scanning direction as the tip–surface distance decreases. This phenomenon implies a spatial expansion of the high dissipation areas (ridges).

**Figure 2:**
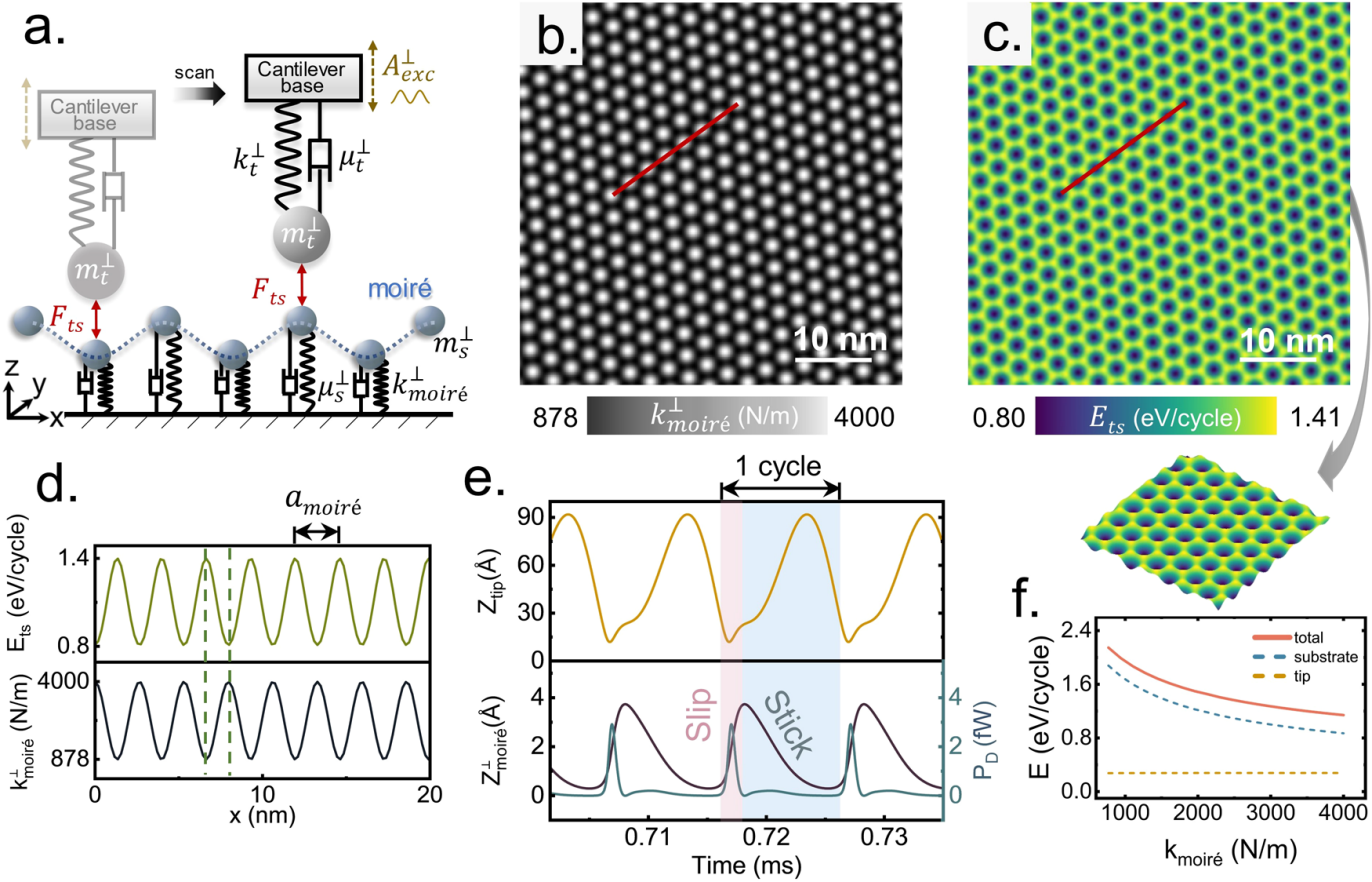
Simulation of moiré vertical dissipation. (a) Schematics of the DFM model. (b) Vertical stiffness map of moiré superlattices, and (c) corresponding dissipation map of the system, inset showing an enlarged 3D view of the dissipation. (d) Line profile extracted from the red lines in (b, c). (e) shows the instantaneous tip displacement, moiré vertical deformation and the power dissipation of the system over a period of simulation time at a fixed position. (f) The energy dissipation versus moiré stiffness. The total dissipation of the system is the sum of tip and substrate. Calculation parameters: *f* = 164.733 kHz, *a*_moiré_ = 2.71 nm, *m*_*t*_^⊥^ = 2.932 × 10^–11^ kg, *m*_*s*_^⊥^ = 2.932 × 10^–10^ kg; *k*_*t*_^⊥^ = 31.41 N/m; damping ratio is 1000 for the substrate and 1.26 × 10^–5^ for the tip.

To account for the observed physical phenomena, we developed a dynamic force microscopy (DFM) model to mimic the kinetic process of the tip-moiré system in NC-AFM. As illustrated in Figure 2(a), the DFM model consists of two components, the AFM tip and the moiré superstructure, which interact through attractive vdW forces *F_ts_*. The tip is driven by an external sinusoidal excitation to compensate the mechanical dissipation of the system. The dynamics of tip and substrate can be described by the equation of motion
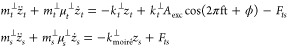
1The superscript ⊥ indicates vertical direction of motion. The subscript “*t*” refers to the tip, and “*s*” indicates the moiré sample. The *m*_t_^⊥^, *k*_*t*_^⊥^, *m*_*s*_^⊥^, *k*_moiré_^⊥^ are the effective mass and spring constant of the tip and the moiré, respectively. The damping coefficients of the tip apex and the substrate are represented by μ_*t*_^⊥^ and μ_*s*_^⊥^. *z*_*t*_ and *z*_*s*_ are the vertical positions of the tip and the substrate, respectively. In the simulations, the free tip is initially excited at the first flexural resonance frequency *f* with an amplitude *A*_exc_ and a phase shift of 90°, which corresponds to the experimental settings. The interaction force between AFM tip and sample gives rise to extra dissipation, which is compensated by the excitation voltage to keep the cantilever amplitude constant at 4 nm. In the calculation the tip–sample distance is not regulated as it varies minimally. All subsequent results presented are obtained under these stabilized conditions with amplitude feedback engaged, unless stated otherwise. The parameters in the calculation are set experimentally, including resonance frequency, cantilever stiffness, target excitation amplitude, etc. More details of the DFM model simulation can be found in Section 3 of the SI.

A higher vertical elasticity of moiré ridges is to be expected, which is supported by previous density functional theory (DFT) studies revealing the flexibility of graphene on the Ir(111) system^32−34^ and the correlation between topography and binding energy.^35,36^ This is also consistent with our NC-AFM results: the moiré ridge is 250 pm higher than the flat domain (line profile in Figure 1(b)). Thus, in the simulation, the vertical stiffness of the moiré (see Figure 2(b)) is defined according to the hexagonal structure of the moiré superlattice as follows
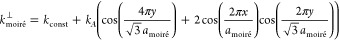
2Where *k*_const_ is the baseline of the substrate stiffness to ensure *k*_moiré_ is always positive. *k*_A_ is the amplitude of *k*_moiré_. The mean vertical dissipation per oscillation cycle of the system includes tip and substrate and can be expressed as
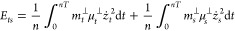
3The DFM model is solved by numerical integration using the Runge–Kutta method. Figure 2(c) shows the simulated vertical dissipation of such a moiré superstructure, which is in good agreement of the experimental dissipation measured by NC-AFM in Figure 1(c). For simplicity, we treat the stiffness of FCC and HCP regions equally in the calculations. A more detailed examination of the dissipation calculations, which also takes into account the stiffness differences between FCC and HCP regions, is presented in Section 3 of the SI. These simulation results suggest that variations in surface stiffness due to the moiré structure significantly influence vertical energy dissipation, with increased stiffness associated with reduced dissipation, as illustrated by the line profiles in Figure 2(d). The overall trend of the energy dissipation of the system as a function of the substrate stiffness is shown in Figure 2(f). In this system, the substrate predominantly governs the energy dissipation, with only a minimal contribution from the probe. Moreover, the softer the substrate, the more significant the dissipation observed. Further insights into this stiffness-dependent vertical dissipation are revealed through an examination of the dynamic response of the substrate under the influence of tip–sample vdW interaction. As the tip oscillates (*Z*_tip_^⊥^, upper panel in Figure 2(e)), the moiré vertical deformation (*Z*_moiré_^⊥^, black line in Figure 2(e) lower panel) shows a sudden upward instability (pink shade) as the tip approaches. Thereafter, a strong tip–substrate interaction remains until the tip retracts, leading to a delayed and slow release (blue shade). This nonlinear dynamic behavior, characterized by an abrupt instability followed by a gradual release, is expected to be closely linked to energy dissipation. The total power dissipation of the system (blue line in Figure 2(e)) reveals that the dissipation primarily occurs during the sudden instability phase (pink shade), while the slow release contributes less. Inspired by the stick–slip concept in nanotribology, we propose this dynamic behavior within an oscillating cycle in NC-AFM as a “vertical stick–slip instability”. Here, the slow detachment of tip and substrate resembles the “stick” phase, while the sudden snap-in due to close proximity (strong vdW force) mirrors the “slip” phase.

Following the identification of the vertical stick–slip instability behavior in the DFM model, it is crucial to analyze the conditions under which energy dissipation occurs due to this instability. By evaluating the interplay between the elastic restoring force of the substrate and the vdW interaction force, we are able to identify the exact conditions that lead to energy dissipation. In our system, dissipation is shown to be dominated by the moiré structure, thus we focus our force analysis on the moiré within one oscillating cycle. In the static approximation, a local extremum (maximum or minimum) of substrate potential *V*^⊥^ is given by the equilibrium force condition (∂*V*^⊥^/∂*z*_*s*_ = 0) leading to
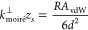
4where tip–sample distance *d* = *z*_*t*_ – *z*_*s*_. *R* is radius of tip apex, *A*_vdW_ is Hamaker constant. When the force gradient becomes negative, there is a transition from a locally stable state (∂^2^*V*^⊥^/∂*z*_*s*_^2^ > 0) to an unstable state (∂^2^*V*^⊥^/∂*z*_*s*_^2^ ≤ 0), corresponding to the occurrence of vertical instability. Consequently, the condition for vertical instability can be characterized by
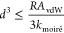
5During a single oscillation cycle: As the distance between tip and sample is large, the vdW force is initially relatively weak. However, as the tip continues its downward motion during oscillation, the attractive vdW force increases. At a critical point, the vdW force exceeds the elastic restoring force of the substrate. When the overall force gradient of substrate changes from positive to negative, this signifies a transition from a stable to an unstable state. This transition is the point at which vertical instability is triggered, resulting in energy dissipation. According to the derived equation eq 5, once the properties of the tip and the surface are determined, dissipation occurs exclusively with respect to the tip–sample distance. As soon as the distance exceeds a critical value, the substrate enters an unstable state—instability, which causes a nonlinear response.

This vertical instability essentially determines the mechanical dissipation in moiré systems. The underlying mechanism of surface-elasticity-dependent dissipation in NC-AFM is therefore attributed to the change in the nonlinear dynamics of the moiré superstructures, which is influenced by the heterogeneous stiffness across the moiré ridges and the flat domains. The noncontact AFM results reveal that the vertical dissipation within the moiré superstructures reflects the variation of the tip–sample interaction with the superlattice, with significant enhancement observed at the moiré ridges. Our DFM model calculations suggest that the dissipation is primarily driven by the vertical stick–slip instability induced by the attractive tip–sample force. The anisotropy in vertical elasticity inherent to the moiré surface microstructure modulates the dynamic behavior of this vertical stick–slip motion, thereby accounting for the observed differences in vertical dissipation between the moiré ridges and flat domains.

**Figure 3:**
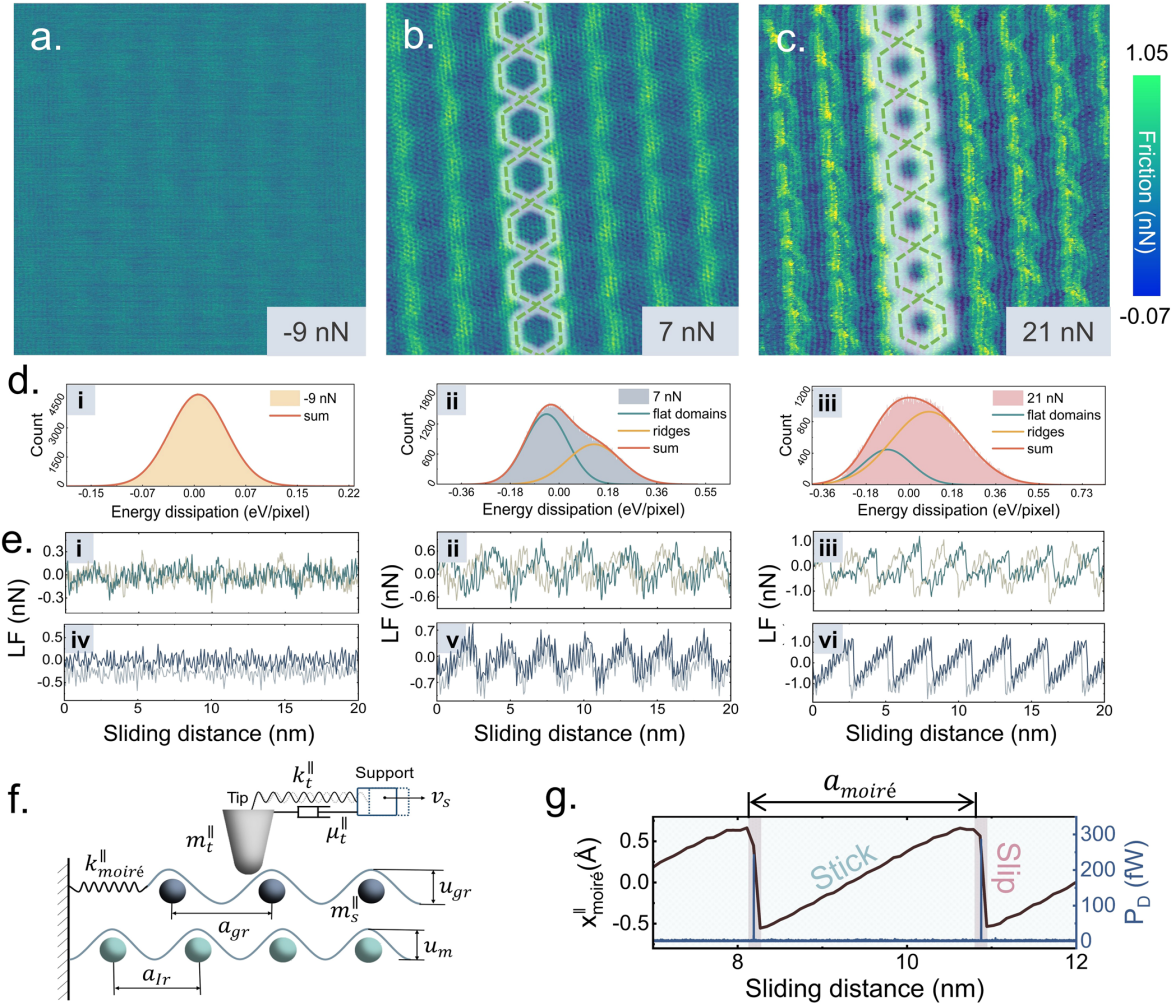
(a–c) Friction force maps of the moiré superstructure under normal loads of −9, 7, and 21 nN, respectively. In addition to the atomic stick–slip, enhanced frictional dissipation patterns of the moiré occur with increasing normal load. Measurement parameters: *v_s_* = 781.3 nm/s, scan size = 20 × 20 nm^2^. (d,i-iii)Histograms of the dissipation distributions from the dissipation maps shown in (a–c), fitted with Gaussian function. (e) Friction loops corresponding to each of the three normal loads. (i–iii): extracted from experiments; (iv–vi): from calculations. Darker lines indicate scanning forward and lighter represent backward. (f) Schematic diagram showing a two-state PF in which a tip slides on a composite structure comprising two periodic layers of monolayer graphene and Ir(111). (g) Power dissipation of the system and the moiré lateral deformation as a function of the sliding distance of the support. Calculation parameters: *v*_s_ = 781.3 nm/s, *k*_*t*_^∥^ = *k*_moiré_^∥^ = 163.1 N/m, *m*_*t*_^∥^ = 2.342 × 10^–10^ kg, *m*_*s*_^∥^ = 2.342 × 10^–10^ kg, *a_gr_* = 0.246 nm, *a*_*Ir*_ = 0.271 nm. (e,iv)*u*_*gr*_ = 0.06 nN·Å, *u*_*m*_ = 0.1 nN·Å; (e,v)*u*_*gr*_ = 0.11 nN·Å, *u*_*m*_ = 1.6 nN·Å; (e,vi)*u*_*gr*_ = 0.15 nN·Å, *u*_*m*_ = 4.15 nN·Å.

Considering the three-dimensional nature of the moiré superstructure, its in-plane elasticity can also play a role in energy dissipation. To study the lateral energy loss in moiré superlattices FFM is applied. In these contact conditions, the tip directly engages against the surface, sliding and dragging the moiré laterally. Figure 3 shows the friction force maps of the moiré surface under normal loads of −9, 7, and 21 nN. Notably, the minus sign of the load indicates that the tip works at the negative tensile force regime to minimize the contact force without detaching from the moiré superstructure. In this case, smooth sliding with ultralow friction between the tip and the moiré is observed at both the atomic scale and the larger moiré scale (see Figure 3(a)), which is confirmed by the friction loop with negligible hysteresis between trace and retrace (Figure 3(e,i)). However, under higher loads, clear dissipative patterns are found with a periodicity of the moiré superlattice. In the friction loops (see Figure 3(e,iii)), a notable hysteresis of the stick–slip at the moiré-scale can be recognized. Specifically, at a normal load of 7 nN, the hexagonal moiré superstructure is already observed, showing a larger friction at the ridges (see Figure 3(b)). The dissipative region, corresponding to higher friction, is further extended when the external load is increased to 21 nN. The observation can be understood by the fact that a lateral elastic deformation of the moiré ridge caused by the tip is enhanced under normal load.^13,14,16^ A weaker binding is found at the moiré ridge,^35,36^ where an elastic deformation can easily be induced by a laterally pushing tip, while the flat graphene domains are more strongly anchored to the substrate and therefore do not deform. This is consistent with our NC-AFM results in Figure 1(b) where a distortion was observed when the tip oscillated close to surface.

Similar to the data from the NC-AFM measurements, the friction dissipation in contact mode shown in Figure 3(a–c) was statistically analyzed, and the corresponding histograms are shown in Figure 3(d,iii). The energy dissipation per pixel was calculated as the product of the friction force and the pixel length. The observation of the energy dissipation distribution on the moiré superstructure from noncontact and contact measurements is in good qualitative agreement. With increasing normal load, which corresponds to stronger tip–sample interactions, the energy dissipation increases, as indicated by the overall rightward shift of the histograms. Furthermore, the contribution from the moiré ridges also becomes more pronounced (as indicated by the yellow solid lines in Figure 3(d)). Considering the spatial extent of the moiré ridges and the flat domains, we roughly calculated the average energy losses per moiré supercell at ridges and flat domains with values of 0.75 and 0.25 eV, 4.43 and 2.87 eV, and 7.74 and 3.99 eV at normal loads of −9, 7, and 21 nN, respectively. The lateral dissipation at the contacting area between the tip and the moiré superstructure remain ultralow as one would expect for superlubric sliding.^37,38^ Notably, the contact dissipation within the superlubric regime is even lower than the noncontact dissipation, while the latter is 1.08 and 0.79 eV/cycle for moiré ridges and flat domains, respectively, at a frequency shift of Δ*f*_1st_ = −70 Hz. In general, vertical and lateral energy dissipation are of the same magnitude on the moiré superstructure. An enhanced dissipation is consistently observed at the ridges in both directions.

To unravel the origin of frictional response of the moiré superstructure, we used a two-state phononic friction (PF) model, which incorporates lateral deformations of the moiré.^16,39^ In the model, an AFM tip is driven by a virtual support via a spring and slides over a sinusoidal potential energy landscape, see Figure 3(f). The top potential *V*_1_ with a graphene lattice constant of *a*_*gr*_ and an amplitude of *u*_*gr*_ describes the tip–surface interaction, which is superimposed by a bottom potential *V*_2_ representing the graphene-Ir(111) interaction, with an Ir(111) periodicity of *a*_*Ir*_ and an amplitude of *u*_*m*_. The stiffness *k*_*t*_^∥^ denotes the effective lateral spring constant of the cantilever and *k*_moiré_^∥^ represents the effective stiffness of the local elastic deformation of the upper graphene moiré superstructure. As the cantilever moves at a constant velocity of *v*_*s*_, the tip and the graphene supercell are displaced by *x*_*t*_ for the tip and *x*_*s*_ for the moiré in-plane deformation. The total potential *V* of the moiré friction system tip-graphene-Ir(111), including the potential interaction tip-graphene, graphene-Ir(111), the spring potential energy for the torsion of the cantilever and the elastic strain energy of the moiré springs, is given by
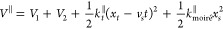
6The dynamics of the system can be described by the Langevin equation of motion (see SI for more details on the two-state PF model)
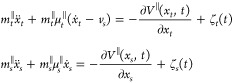
7The numerical calculation results of eq 7 were solved using the fourth-order Runge–Kutta algorithm. As previously discussed, the experimentally measured friction loops in Figure 3(e) show a transition from continuous sliding to a moiré-level stick–slip motion at increasing normal loads. Accordingly, we increase the corrugation heights *u*_*gr*_ and *u*_*m*_ of the potentials to mimic the increasing normal load. As a result, the calculated friction loops (Figure 3(e,iv–vi)) not only represent the transition from superlubric sliding to highly dissipative moiré-level stick–slip qualitatively, but also the absolute values of the friction forces, which quantitatively agree well with the experiment.

In principle, the underlying physical mechanisms of energy dissipation in the Gr/Ir(111) moiré composite system could be elucidated by analyzing the power dissipation together with the in-plane deformation of the moiré superstructure. As shown in Figure 3(g), we extract the calculated moiré lateral displacement (*x*_moiré_^∥^) and power dissipation data with the parameters of Figure 3(e). The solid black line of the moiré deformation in Figure 3(g) shows that during the initial in-plane stretching of graphene, the ridges are pushed forward by the tip, leading to the accumulation of elastic deformation in the moiré. During this process, the tip remains attached to the moiré, and is therefore referred as the stick state^13^ (blue shadow in Figure 3(g)). As soon as the tip overcomes the energy barrier of the interaction between tip and ridge, the ridge destabilizes and detaches from the tip, causing the strain to abruptly decrease and the moiré to retract to its equilibrium position, the slip state (pink shadow in Figure 3(g)). The lateral attachment and detachment motion form together a complete stick–slip period on the moiré-scale. As shown in the right axis of Figure 3(g), the energy is dissipated mainly at the moment of slip instability during the periodic oscillation of the top layer graphene, which corresponds experimentally to the instantaneous release of elastic energy at the moiré ridges. Notably, we do not observe significant peaks of power dissipation in the period of the graphene lattice constant. This suggests that the dissipation contribution from atomic stick–slip is negligible relative to the stick–slip at the moiré-level.

**Figure 4:**
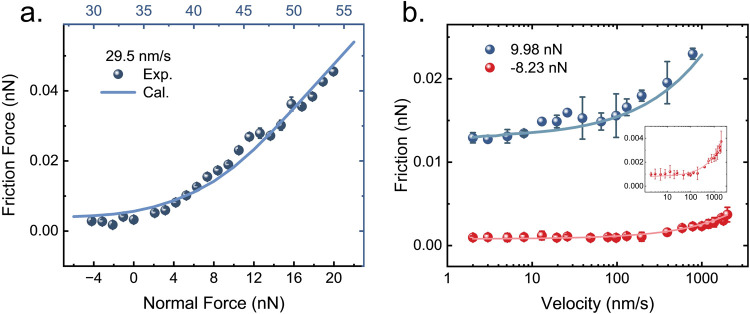
(a) Normal load dependence of friction on the Gr/Ir(111) moiré superlattice at a sliding velocity of 29.5 nm/s and the corresponding calculated result by the two-state PF model. (b) Velocity dependence of the friction at normal loads of 9.98 and −8.23 nN, and the corresponding calculated results. Insets: Enlarged view of the lower curve.

Since the moiré-scale stick–slip is associated with the dynamic processes of the tip-moiré interface, external conditions such as normal load and velocity can be used to tune the friction dissipation of such systems. To this end, the dependence of moiré friction on external load and sliding velocity is systematically investigated. Figure 4(a) shows the load dependence of friction on the moiré. At low normal load (i.e., <0 nN), superlubric sliding with negligible dissipation is observed, corresponding to the case in Figure 3(a). Here, the tip smoothly follows the moiré topological corrugation without moiré lateral deformation. This scenario breaks down as the normal force increases, entering the dissipative regime by a rapid rise in friction. Higher load enhances the surface potential corrugation, resulting in a greater elastic deformation in the moiré ridges due to the pushing tip until a sudden relaxation upon tip sliding. The experimental observation can be well reproduced by the two-state PF model (solid line in Figure 4(a)). The discrepancy of ≈26 nN in normal load between experiment and calculation is considered to be adhesion present in the experiment but not accounted for in the model.

Figure 4(b) presents the friction force as a function of velocity under normal loads of −8.23 and 9.98 nN. In both cases, there is a clear transition between low and almost constant sliding friction at low speeds and a logarithmic scaling of friction at higher velocities. The transition velocity shifts from ≈10 to ≈100 nm/s with decreasing normal load due to a weaker tip-moiré interaction, in agreement with recent experimental observations on the Gr/Pt(111) system.^13^ This involves a competing relationship between two frequency scales, namely the washboard frequency at moiré-level *f*_*wb*_ = *v*/*a*_moiré_ and the frequency of moiré-level stick–slip *f*_*ad*_ (attachment and detachment rate). The former is related to the sliding velocity *v*_*s*_ and the moiré size *a*_moiré_, whereas the latter can be determined by the effective force constant of the moiré system *k*_eff_, which is a combination of the contact stiffness and the moiré lateral deformation stiffness. Consequently, *f*_*ad*_ can be described as . The attachment-detachment rate is therefore mainly determined by the contact stiffness, which can be altered by varying the normal load (corrugated potential in calculation). At low velocities, i.e., *f*_*wb*_ < *f*_*ad*_, each moiré experienced by the tip has enough time to relax gently until the tip reaches the next one. In this case, both the attachment and detachment motions are gentle and barely contribute to frictional dissipation. Yet both continuous sliding and stick–slip are possible at the atomic scale, which will cause a slight change in the slope at low velocities. However, this slight change is difficult to be detected experimentally due to the limitation of instrument sensitivity. At higher speeds, the probe passes rapidly over the moiré superstructure, causing deformation at the moiré ridge, followed by rapid relaxation of the local strain, leading to remarkable friction dissipation. In addition, a higher normal load enhances the energy barrier as well as the moiré strain as the contacting atoms are pressed closer to the moiré ridge, both of which lead to a greater dissipation even at a relatively low velocity. This is the reason why the threshold velocity shifts toward a smaller value under the higher load. The two-state PF model featured the moiré-level stick–slip dynamics well and reproduces our experimental observations of the frictional velocity dependence between the sliding tip and the moiré superstructure.

## Conclusions

In summary, we have systematically investigated the energy dissipation mechanisms of the moiré superstructure, emphasizing its nonlinear dynamic processes. Our NC-AFM and FFM studies show an enhanced dissipation at the graphene moiré ridges compared to the flat domains, a discrepancy related to the different mechanical responses and inherent elasticity of these structures. Specifically, moiré ridges, with their higher elasticity, exhibit pronounced vertical and lateral stick–slip instabilities, identified as the fundamental cause of mechanical energy dissipation. A DFM model is developed and marks a significant advancement in simulating NC-AFM dissipation processes, while a two-state PF model is employed for FFM studies. Integrating these models, we established a unified phononic dissipation framework that not only links dissipation to vibrational behaviors of the moiré but also enhances our understanding of energy dissipation across different directional domains. Furthermore, using the insights from the DFM model, we propose specific conditions for the occurrence of a vertical instability in the moiré structure—indicative of energy dissipation—to occur, thus improving the predictive capabilities for the system dissipation behavior under different operational scenarios.

It is important to note that lateral-normal coupling^40^ exists in both NC-AFM and FFM measurement. In NC-AFM, lateral dissipation is negligible, while in FFM, vertical dissipation increases with normal load but remains minor overall. Despite this coupling, dissipation is still dominated by the primary driving direction. Consequently, we treated normal and lateral motion independently in our models. Additionally, the DFM model overestimates vertical deformation due to its simplified treatment of substrate stiffness, which neglects lateral interatomic coupling. By modeling each atom as an independent oscillator, the model amplifies vertical displacements that would be suppressed in a real system with lateral constraints.

Building on the insights from our experimental and modeling efforts, we can outline effective strategies for managing mechanical energy dissipation in moiré structures. Key findings indicate that an effective reduction of energy dissipation in both the vertical and lateral directions can be achieved by the increase of moiré stiffness, using smaller moiré sizes, or enhancing substrate interactions. Once the contact moiré system is determined, increasing the separation between the moiré and contact bodies reduces vertical dissipation, while appropriate regulation of the normal load can achieve superlubricity, minimizing lateral dissipation. Additionally, lowering the sliding velocity between contact surfaces significantly reduces nonlinear dynamic responses, thereby reducing energy dissipation. Our detailed investigation of dissipation mechanisms provides a fundamental understanding necessary for controlling energy dissipation in the design of devices with moiré-based components.

## Methods

All the experiments are carried out with a home-built UHV AFM at room temperature. Sample preparation is performed in a preparation chamber with a base pressure in the range of 10^–10^ mbar. Subsequently, the sample was transferred to the analytical chamber for AFM measurements with a better base vacuum below 1 × 10^–10^ mbar. The single crystal Ir(111) sample (MaTeck GmbH, Germany) was treated by repeated cycles of *Ar*^+^ sputtering with the power of 1.8 keV at a chamber pressure of 3 × 10^–6^ mbar for 10 min, and then annealing at 1450 K for 30 min by a homemade radio frequency (RF) heater. Fully covered monolayer graphene is subsequently synthesized on the clean Ir(111) surface by dosing C_2_H_4_ gas at partial pressure of 5 × 10^–8^ mbar for 1 min at 1400 K, followed by annealing at the same temperature for 3 more minutes.

Two different cantilevers (Nanosensor) were used in the NC-AFM and FFM experiments, PPP-NCL for the former and PPP-CONT for the latter. Both of the cantilevers are prepared with the same procedure: annealed in UHV at temperature of 150 °C for 1 h to remove impurities from the air, and followed by *Ar*^+^ sputtering at 800 eV for 2 min to remove the naturally formed silicon dioxide on the tip. Before the experiment, the stiffness and sensitivities of both cantilevers are calibrated in vertical and lateral directions.

## Data Availability

The raw data used in this study are available from Zenodo.^41^

## Supporting Information Available

Details of energy dissipation in dynamic force microscopy, details for the DFM model and the two-state PF model calculation (PDF)
