# Supplementary material for: Moiré Energy Dissipation Driven by Nonlinear Dynamics
Source: ACS Nano. 2025 Apr 30;19(18):17365–73. doi: 10.1021/acsnano.4c16817 (PMC12080335; doi:10.1021/acsnano.4c16817)
Supplement: Supplementary file 1 — nn4c16817_si_001.pdf [file nn4c16817_si_001.pdf]

Moiré energy dissipation driven by nonlinear dynamics

Shuyu Huang,[†,‡] Yiming Song,[*,¶] Antoine Hinaut,[‡] Gema Navarro-Marín,[‡] Yunfei Chen,[*,†] Ernst Meyer,[‡] and Thilo Glatzel[*,‡]

†*Key Laboratory for Design and Manufacture of Micro-Nano Biomedical Instruments, School of Mechanical Engineering, Southeast University, Nanjing 211189, China*

‡*Department of Physics, University of Basel, 4056 Basel, Switzerland*

¶*Institute for Applied Physics, Justus Liebig University Giessen, 35392 Giessen, Germany*

E-mail: songym_thu@yeah.net; yunfeichen@seu.edu.cn; thilo.glatzel@unibas.ch

1. Energy dissipation in non-contact AFM experiments

(a) Vertical dissipation

In NC-AFM measurements, in order to maintain the amplitude of the tip oscillations, an additional sinusoidal energy input signal, called 'excitation' V_{exc}, must be constantly applied to the cantilever. The influence of electrostatic forces between the AFM probe and the substrate is eliminated by applying an external DC bias voltage to compensate the contact potential difference. Thus, the excitation is related to the energy dissipation induced by the tip-surface interaction, which varies with the tip-surface distance (frequency shift f in experiments).[1,2]

Before calculating the energy dissipation due to the tip-surface interaction, the power dissipation P_0 arising from the internal damping of cantilever should be estimated with the

following formula:

$$P_0 = 2\pi f_0 \frac{kA_0^2}{2Q}. \quad (1)$$

The subscript '0' denotes a freely oscillating cantilever, meaning that the effects of tip-surface interactions are not involved. Therefore, f_0 and A_0 are the first flexural resonance frequency and the amplitude of the freely oscillating cantilever, respectively. Q is the quality factor of the oscillation, while k is the spring constant of the cantilever in vertical direction.

The dissipation P_0 is due to internal friction, and is independent of the sample and unavoidable. Upon scanning, additional power may be dissipated by the tip-sample interaction and can be calculated from P_0 and the excitation V_{exc}:

$$P = P_0(\frac{V_{exc}}{V_{exc,0}} - 1). \quad (2)$$

Where V_{exc} is the excitation voltage signal during scanning, and $V_{exc,0}$ stands for the excitation voltage required to maintain the oscillation amplitude when the tip is oscillating freely far from the sample.

The energy dissipation per oscillating cycle can be obtained from the power dissipation P and the actual oscillation frequency, which is calculated as the sum of resonance frequency f_0 and the real-time frequency shift Δf during scanning:

$$E = P \cdot \frac{1}{f_0 + \Delta f}. \quad (3)$$

In order to calculate the energy dissipation in NC-AFM from the eq. 1 to 3, the excitation and frequency shift signals are required. The original excitation voltage V_{exc} recorded in the measurements are shown in Fig. S1 (a) and (b) with the set frequency shift of -70 Hz and -104 Hz, respectively. The instantaneous frequency shift map Δf (Fig. S1 (c) and (d)) indicated that the desired constant frequency shift is well controlled by the phase-locked loop during scanning.

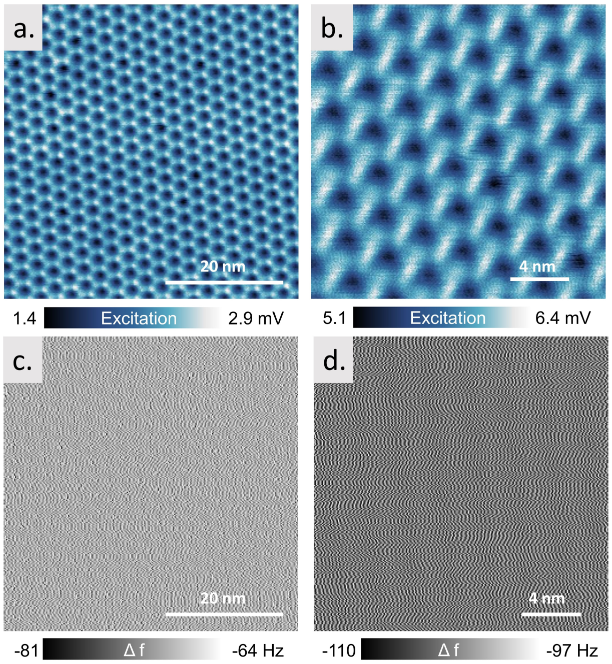

Figure S1: (a-b) The original excitation signals with setpoint of -70 Hz and -104 Hz, respectively. (c-d) The corresponding instantaneous frequency shift signals.

Besides V_{exc} and Δf, the values of the remaining parameters in our experiments are given below: f_0=164.733 kHz, k=31.4 N/m, $A = 4$ nm, Q=39543, $V_{exc,0} = 457\mu$V.

(b) Lateral dissipation and lateral force

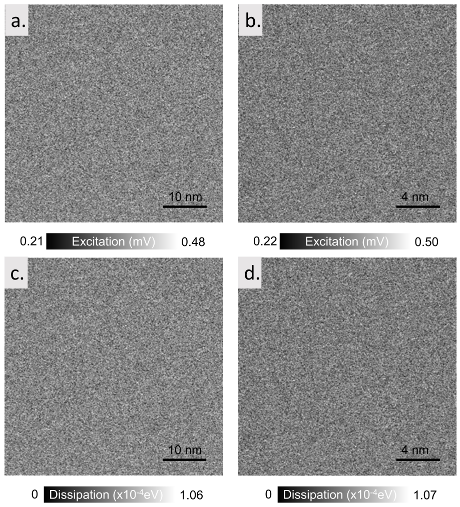

Figure S2: (a-b) The torsional excitation signals with a frequency shift setpoint of -70 Hz and -104 Hz, respectively. (c-d) The corresponding derived dissipation maps.

In our experiment, the cantilever does not only oscillate at the first flexural resonance f_{1st}

but also simultaneously parallel to the surface at the first torsional resonance f_{TR} (named bimodal NC-AFM). Hence, lateral dissipation in NC-AFM can be derived from torsional excitation signal with the same method described in eqs. 1 to 3 by using the parameters specific to lateral direction. The torsional excitation and corresponding lateral dissipation is shown in Fig. S2.

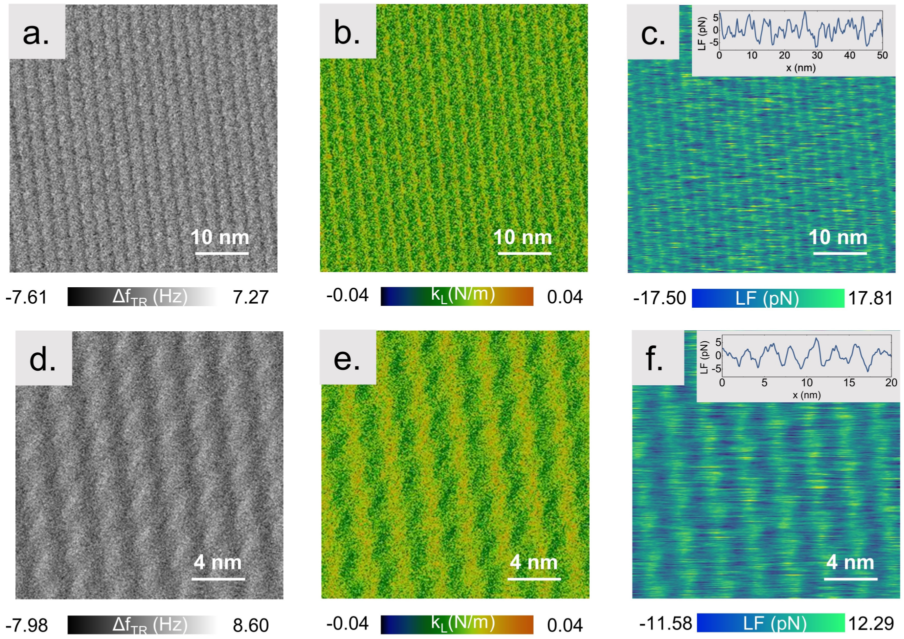

Figure S 3: The torsional frequency shift, corresponding lateral-force gradient maps and lateral forces with a setpoint of (a-c) -70 Hz and (d-f) -104 Hz, respectively.

Notably, no moiré dissipation contrast is observed in lateral direction, and the energy dissipation is nearly zero at both tip-sample distances. Besides, the torsional frequency shift Fig. S3(a,d) can be regard as lateral-force gradient (see Fig. S3(b,e)), and thus lateral force in NC-AFM can be numerically obtained as,[3,4]

$$F_L(x) = \int_{x_{F_x=0}}^{x} -\frac{2k_{TR}\Delta f_{TR}}{f_{TR}} dx, \quad (4)$$

where x is the tip displacement in lateral direction. k_{TR}, f_{TR}, Δf_{TR} are cantilever stiffness, resonance frequency and frequency shift in torsional direction, respectively. The converted lateral force could be atomically resolved with resolutions of below 1 pN in the non-contact

region, as shown in Figs. 3 (c) and (f) at frequency shift of -70 Hz and -104 Hz, respectively. The non-contact lateral force are found to be ±5 pN and are greater at the elastic moiré ridges.

2. The dynamic force microscopy (DFM) model

The DFM is developed to simulate dynamics of both probe and surface in non-contact AFM, both the AFM probe and the moiré substrate are modeled with a damped harmonic oscillator, as shown in Fig. S4(a). The dynamics of tip and substrate can be described by the equation of motion

$$\begin{aligned} m_t^{\perp}\ddot{z}_t + m_t^{\perp}\mu_t^{\perp}\dot{z}_t &= -k_t^{\perp}z_t + k_t^{\perp}A_{exc}cos(2\pi ft + \phi) - F_{ts}(z_t - z_s); \\ m_s^{\perp}\ddot{z}_s + m_s^{\perp}\mu_s^{\perp}\dot{z}_s &= -k_{moir\acute{e}}^{\perp}z_s + F_{ts}(z_t - z_s), \end{aligned} \tag{5}$$

where z is the vertical position and f is the first flexual resonance frequency of the cantilever. $m_t^{\perp}$, $k_t^{\perp}$, $m_s^{\perp}$, $k_{moir\acute{e}}^{\perp}$, are the effective mass and vertical stiffness of the tip and the moiré, respectively. $k_t^{\perp}$ is calibrated to be 31.41 N/m and $m_t^{\perp}$ is determined from $k_t^{\perp}$ and f following: $m_t^{\perp} = k/(2\pi f)^2$. $m_s^{\perp}$ is estimated 10 times greater than $m_t^{\perp}$. The energy of the system input is simulated by a sinusoidal excitation signal from the cantilever base, which is expressed as: $A_{exc}cos(2\pi ft)$. The excitation has a 90° phase ϕ difference compared to the cantilever oscillation. The energy transfer to the substrate is included in the tip-sample interaction force F_{ts} and dissipated through the dampers in both tip and substrate. Note that since the Q factor of the freely oscillating cantilever under ultra-high vacuum in our experiment is 39543 with a resonant frequency of 164.733 kHz, the internal friction of the cantilever is considered to be extremely low, i.e. highly underdamped. The damping ratio of the tip is set to be 1.26×10^{-5}, which is determined by the Q factor, the stiffness and the resonance frequency from the experiment. The substrate is considered to be overdamped with a damping ratio of 1000. Therefore, the damping coefficients are given as: $\mu_t^{\perp} = 1.26 \times 10^{-5} \times 2\sqrt{k_t^{\perp}/m_t^{\perp}}$

for the tip, and $\mu_s^\perp = 1000 \times 2\sqrt{k_2^\perp/m_s^\perp}$ for the substrate. Since the tip oscillates in the attractive force regime in NC-AFM, the tip-sample interaction is described as an attractive vdW force, $F_{ts} = -\frac{A_{vdW} R}{6(z_t - z_s)^2}$. R is the radius of the tip, which is set as 10 nm. A_{vdW} is the Hamaker constant, we use 1×10^{-15} J in the calculation. $(z_t - z_s)$ represent the tip-sample distance. Attention should be paid to the fact that the maximum spring force should be greater than the vdW force, both in the tip and in the substrate case, to avoid oscillation stopping.

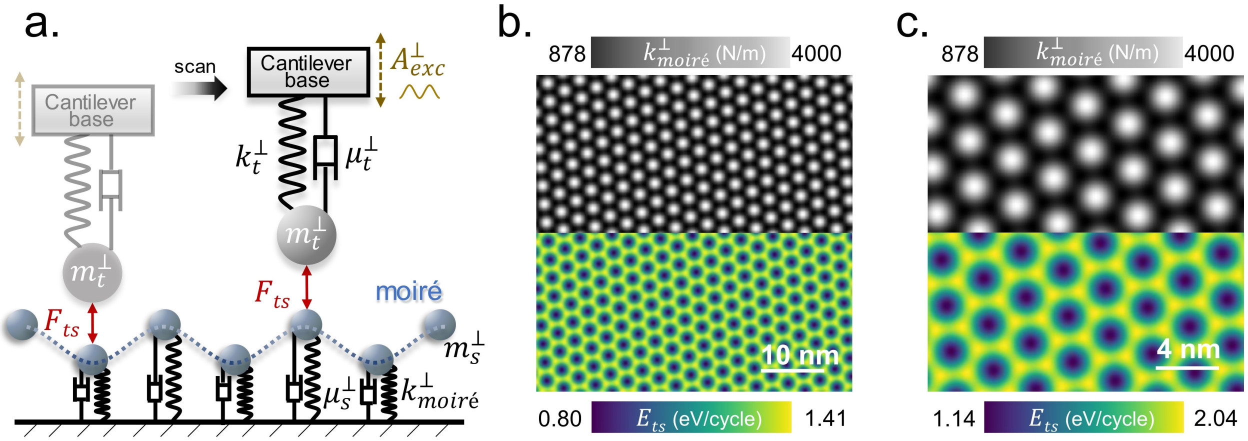

Figure S4: (a) Schematics of the DFM model. (b) The substrate vertical stiffness and energy dissipation per cycle of the system with a scan size of 50 nm and tip-sample distance of 17.6 nm, and (c) with a scan size of 20 nm and tip-sample distance of 5.1 nm.

The model is numerically solved by a fourth-order Runge–Kutta algorithm. There are three states in each simulation: First: the tip starts by being excited at a target amplitude of 4 nm without interacting with the sample (i.e. $F_{ts} = 0$) until the oscillation stabilizes. This is used to calculate the excitation amplitude required for the tip to oscillate freely. Second: tip-sample interaction forces are introduced, causing the amplitude to decrease and waiting for the system to stabilize again. Third state: Amplitude feedback is activated to restore the amplitude at the desired 4 nm amplitude by adjusting the excitation amplitude (input energy). All results presented are obtained under these stabilized conditions with amplitude feedback engaged, unless stated otherwise.

The experimental results presented in Fig. 1 of the main text reveal the presence of the

three high-symmetry domains—ATOP, FCC, and HCP—within the moiré superstructure, as observed in both topography and dissipation analyses. For simplicity, the vertical stiffness of the FCC and HCP regions is initially assumed to be identical. However, the variations in binding energy between the FCC and HCP domains can subtly influence the local stiffness, resulting in changes in energy dissipation. This difference can also be incorporated into the model by introducing a modulation term to the existing stiffness, which is described as follows:

$$k_{mod}^{\perp} = -k_{A,2}(cos(\frac{4\pi(y-\frac{\sqrt{3}}{3}a)}{\sqrt{3}a_{moiré}}) + 2cos(\frac{2\pi x}{a_{moiré}})cos(\frac{2\pi(y-\frac{\sqrt{3}}{3}a)}{\sqrt{3}a_{moiré}})). \tag{6}$$

where $k_{A,2}$ represents the amplitude of stiffness modulation. Consequently, the total potential energy, considering differences in FCC and HCP stiffness, is expressed as $k_{mod}^{\perp} + k_{moiré}^{\perp}$. The resulting vertical stiffness and corresponding dissipation are presented in in Fig. S5, which closely aligns with the dissipation characteristics of the three regions observed experimentally.

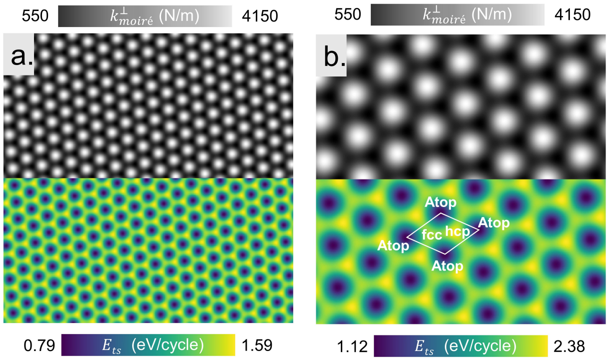

Figure S 5: Considering variations in vertical stiffness across ATOP, FCC, and HCP regions:(a) the substrate vertical stiffness and energy dissipation per cycle of the system maps with a scan size of 50 nm and tip-sample distance of 17.6 nm, and (b) with a scan size of 20 nm and tip-sample distance of 5.1 nm.

3. The two-states PF model calculation

In the Langevin equation of the two-states PF model, μ_t and μ_s are the equivalent damping coefficients describing the dissipation from the tip-graphene and graphene-Ir(111) interface, repetitively. We consider μ in all our cases as critical damping. The thermal noise term $\zeta_t(t)$

and $\zeta_s(t)$ are the random effects of fluctuation satisfying the fluctation-dissipation relation: $\langle\zeta_i(t)\zeta_i(t')\rangle = 2m_i\mu_i k_B T\delta(t-t')$, where k_B is the Boltzmann constant and T is the absolute temperature.

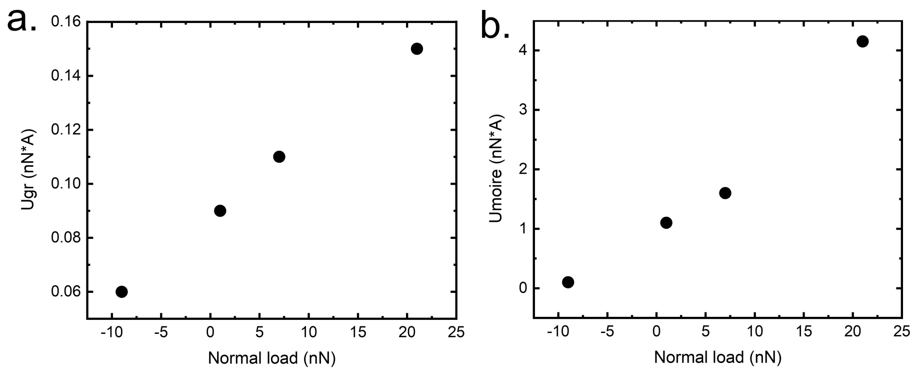

Figure S 6: Corrugation amplitude of the (a) tip-graphene interaction potential and (b) graphene-Ir(111) interface interaction as a function of the normal load.

The amplitudes of the corrugated potential for both the tip-graphene and the graphene-Ir(111) interfaces in the calculations are proportional to the normal load in the experiments, as shown in Fig. S6.

References

(1) Loppacher, C.; Bennewitz, R.; Pfeiffer, O.; Guggisberg, M.; Bammerlin, M.; Schär, S.; Barwich, V.; Baratoff, A.; Meyer, E. Experimental Aspects of Dissipation Force Microscopy. *Physical Review B* **2000**, *62*, 13674–13679.

(2) Meyer, E.; Bennewitz, R.; Hug, H. J. *Scanning Probe Microscopy: The Lab on a Tip*; Graduate Texts in Physics; Springer International Publishing: Cham, 2021.

(3) Sader, J. E.; Jarvis, S. P. Accurate Formulas for Interaction Force and Energy in Frequency Modulation Force Spectroscopy. *Applied Physics Letters* **2004**, *84*, 1801–1803.

(4) Kawai, S.; Sasaki, N.; Kawakatsu, H. Direct Mapping of the Lateral Force Gradient on Si 7x7. *Physical Review B* **2009**, *79*, 195412.
